# *Eimeria bovis* infection modulates endothelial host cell cholesterol metabolism for successful replication

**DOI:** 10.1186/s13567-015-0230-z

**Published:** 2015-09-23

**Authors:** Penny H. Hamid, Joerg Hirzmann, Katharina Kerner, Gerald Gimpl, Guenter Lochnit, Carlos R. Hermosilla, Anja Taubert

**Affiliations:** Institute of Parasitology, Biomedical Research Centre, Justus Liebig University Giessen, Schubertstr. 81, D-35392 Giessen, Germany; Institute for Hygiene and Infectious Diseases of Animals, Justus LiebigUniversity Giessen, Frankfurter Str. 85-89, D-35392 Giessen, Germany; Institute of Pharmacy and Biochemistry, Department of Biochemistry, Johann-Joachim-Becherweg 30, D-55099 Mainz, Germany; Institute of Biochemistry, Justus Liebig University Giessen, Friedrichstr. 24, D-35392 Giessen, Germany

## Abstract

During first merogony *Eimeria bovis* forms large macromeronts in endothelial host cells containing >120 000 merozoites I. During multiplication, large amounts of cholesterol are indispensable for the enormous offspring membrane production. Cholesterol auxotrophy was proven for other apicomplexan parasites. Consequently they scavenge cholesterol from their host cell apparently in a parasite-specific manner. We here analyzed the influence of *E. bovis* infection on endothelial host cell cholesterol metabolism and found considerable differences to other coccidian parasites. Overall, free cholesterol significantly accumulated in *E. bovis* infected host cells. Furthermore, a striking increase of lipid droplet formation was observed within immature macromeronts. Artificial host cell lipid droplet enrichment significantly improved *E. bovis* merozoite I production confirming the key role of lipid droplet contents for optimal parasite proliferation. The transcription of several genes being involved in both, cholesterol de novo biosynthesis and low density lipoprotein-(LDL) mediated uptake, was significantly up-regulated at a time in infected cells suggesting a simultaneous exploitation of these two cholesterol acquisition pathways. *E. bovis* scavenges LDL-derived cholesterol apparently through significantly increased levels of surface LDL receptor abundance and LDL binding to infected cells. Consequently, LDL supplementation significantly improved parasite replication. The up-regulation of the oxidized LDL receptor 1 furthermore identified this scavenger receptor as a key molecule in parasite-triggered LDL uptake. Moreover, cellular cholesterol processing was altered in infected cells as indicated by up-regulation of cholesterol-25-hydroxylase and sterol O-acyltransferase. Overall, these results show that *E. bovis* considerably exploits the host cell cholesterol metabolism to guarantee its massive intracellular growth and replication.

**Electronic supplementary material:**

The online version of this article (doi:10.1186/s13567-015-0230-z) contains supplementary material, which is available to authorized users.

## Introduction

*Eimeria bovis* represents one of the most pathogenic *Eimeria* species causing cattle coccidiosis [1]. During its long-lasting intracellular first merogony (14–18 days of duration) *E. bovis* forms large macromeronts of up to 400 μm in size containing ≥120 000 merozoites I within a parasitophorous vacuole (PV) in host endothelial cells [2]. Given that the invading sporozoite stage alone cannot provide all components necessary for this nutrient and energy demanding process, the parasite needs to scavenge molecules from the host cell. Especially for the offspring membrane biosynthesis, large amounts of cholesterol are indispensable for a successful replication process.

Coccidian parasites have been described as auxotrophic in their capability to synthesize cholesterol by themselves [3–9]. Thus, cholesterol auxotrophy was reported for *Toxoplasma gondii* and *Cryptosporidium parvum* [4,7,9]. In consequence these parasites scavenge cholesterol from their host cell thereby exploiting different cellular pathways. Interestingly, different cholesterol auxotrophic parasites appear to follow different strategies of cholesterol scavenge suggesting the modulation of host cell cholesterol metabolism as a parasite-specific and/or host cell-specific process. Coppens et al. [4] experimentally proved *T. gondii* as deficient in cholesterol biosynthesis and showed that this parasite scavenges cholesterol from its host cell. However, different strategies were described in different *T. gondii*-infected host cell types. Thus, in contrast to macrophages [10], *T. gondii* exclusively utilized cholesterol derived from internalized LDL particles in CHO cells [4] whilst transcriptomic data on infected fibroblasts indicated an up-regulation of molecules being involved in the mevalonate pathway [11]. The pivotal role of cholesterol was also proven by exogenously supplied cholesterol which improved *T. gondii* replication [4]. Cholesterol is inserted in the parasite plasma membrane, the parasitophorous vacuolar membrane (PVM), sequestered in cholesterol-rich organelles in *T. gondii*-infected host cells and is furthermore esterified for lipid droplet deposition [4,12–14]. Accordingly, host and parasite esterification activity was shown to be essential for *T. gondii* intracellular growth [15]. Correspondingly, *T. gondii* infection leads to enhanced cytoplasmic lipid droplet formation in skeletal muscle cell cultures [16].

By far less data on cholesterol scavenge is available from other coccidian parasites. *Cryptosporidium parvum* mainly acquires cholesterol from LDL particles and from micelles being internalized by the infected enterocytes but only to a minor degree from host cellular *de novo*-synthesis [7]. The non-coccidian but apicomplexan parasite *Plasmodium yoelii* in principle salvages cholesterol in infected hepatocytes from both host cellular pathways, [6,17] although host cellular cholesterol acquisition does not appear to be essential for its optimal proliferation [6]. The authors interpret these results by a moderate parasitic need of sterols and by an adaptive reaction to cholesterol-restricted conditions in terms of alternative source utilization. Abundant lipid droplet formation was also reported for *P. berghei*- or *P. falciparum*-infected host cells [18,19], but no cholesteryl ester formation was detected in *Plasmodium*-infected cells, implying a lack of lipid storage activity [19–22] which may argue for a continuous cholesterol acquisition from the host cell as hypothesized by Coppens [9].

To date, no detailed analyses on cholesterol acquisition exist for any *Eimeria* species. All farm animals kept under conventional farming conditions unavoidably are exposed to *Eimeria* spp. infections worldwide [1,23] and infection-induced impaired animal performance, mortality and anticoccidial treatment costs generally result in considerable economic losses [1,24]. As an obligate intracellular protozoa residing within a PV, *Eimeria* spp. require cholesterol not only for PV establishment and host cell (membrane) enlargement (especially in macromeront-forming species) but in particular for massive offspring production (>120 000 merozoites I in case of *E. bovis*). Consequently, global transcriptomic approaches on infected endothelial host cells have indicated that *E. bovis* is in need for cholesterol for its successful replication [25]. In addition, recent inhibitor analyses indicated a pivotal role of the host cellular cholesterol de novo synthesis for parasite replication since treatments of *E. bovis*-infected cells with inhibitors of the mevalonate pathway significantly blocked parasite proliferation [26]. However, detailed analyses on this topic are lacking so far. Therefore we here conducted analyses on *E. bovis*-induced alterations of the mevalonate pathway, on lipid droplet formation and on LDL-mediated cholesterol internalization and showed that apparently this parasite applies comparable but also different strategies compared to other coccidian species to meet its needs for cholesterol for successful macromeront formation.

## Materials and methods

### Parasite

The *E. bovis* strain H used in the present study was initially isolated from the field in Northern Germany [27] and maintained by passages in parasite-free male Holstein Friesian calves [28]. All animal procedures were performed according to the Justus Liebig University Animal Care Committee guidelines, approved by the Ethic Commission for Experimental Animal Studies of the State of Hesse (Regierungspräsidium Giessen, GI 18/10 No A37/2011, JLU-No 494) and in accordance to the current German Animal Protection Laws.

For oocysts production, calves were infected orally at the age of 10 weeks with 3 × 10^5^ sporulated *E. bovis* oocysts. The oocysts were isolated from the faeces beginning 18 days post infection (pi) according to the method of Jackson [29]. The sporulation of oocysts was achieved by incubation in a 2% (w/v) potassium dichromate (Merck) solution at room temperature (RT). Sporulated oocysts were stored in 2% (w/v) potassium dichromate solution at 4 °C until further use. Sporozoites were excysted from sporulated oocysts as previously described [30]. Free sporozoites were washed three times in phosphate buffered-solution (PBS) and counted in a Neubauer chamber.

### Host cells and *Eimeria bovis* host cell infections

Primary bovine umbilical vein endothelial cells (BUVEC) were isolated according to Jaffe et al. [31]. Umbilical cords were collected under aseptic conditions from animals born by *sectio caesaria* and kept at 4 °C in 0.9% HBSS-HEPES buffer (pH 7.4, Gibco) supplemented with 1% penicillin (500 U/mL, Sigma-Aldrich) and streptomycin (50 μg/mL, Sigma-Aldrich) until use. For the isolation of endothelial cells, 0.025% collagenase type II (Worthington Biochemical Corporation) suspended in Pucks solution (Gibco) was infused into the lumen of the ligated umbilical vein and incubated for 20 min at 37 °C in 5% CO_2_ atmosphere. After gently massaging the umbilical vein, the cell suspension was collected and supplemented with 1 mL fetal calf serum (FCS, Gibco) in order to inactivate the collagenase. After two washings (400 × *g*, 10 min, 4 °C), cells were resuspended in complete endothelial cell growth medium (ECGM, PromoCell), plated in 25 cm^2^ tissue plastic culture flasks (Greiner) and kept at 37 °C in 5% CO_2_ atmosphere. BUVEC were cultured in modified ECGM medium (EGCM, PromoCell, diluted 0.3× in M199 medium, Sigma-Aldrich) with medium changes every 2–3 days. BUVEC cell layers were used for infection after 1–2 passages in vitro.

BUVEC layers in 24-well or 25 cm^2^-flask formats were infected at 80–90% confluency with 2 × 10^4^ or 1.5 × 10^6^*E. bovis* sporozoites, respectively. Culture medium was changed 24 h after parasite infection and thereafter every third day. Merozoites I were collected from cell culture supernatants from days 17–20 pi onwards and washed three times in PBS (600 × *g*, 15 min) before further use.

### Staining of cholesterol and lipid droplets

For staining of intracellular stages (1, 8, 14 and 17 days pi), BUVEC were grown on cover slips and infected, whereas invasive stages (sporozoites and merozoites I) were directly dropped onto poly-L-lysine coated coverslips. Specimens were washed with PBS, fixed in 4% paraformaldehyde (10 min), washed three times in PBS and incubated in 1% glycine PBS (10 min) to quench non-specific signals, followed by three washings in PBS. To detect free cholesterol, the samples were stained by filipin (0.05 mg/mL in PBS containing 10% FCS, Sigma-Aldrich, 2 h, in the dark, RT). For neutral lipid/lipid droplet visualization the cells were stained in Nile Red (1:1000, Cayman Chemical, 15 min, RT, in the dark) or with Bodipy 493/503 (1 μg/mL, Life Technologies; 10 min, RT, in the dark). All samples were washed in PBS, mounted in Prolong antifading mounting medium (Life Technologies) and analyzed using an inverted fluorescence microscope or confocal microscope applying the UV filter set for filipin (340–380 nm excitation, 430 nm pass filter) or FITC filter settings for Nile red and Bodipy 493/503. For an alternative LD staining, osmium tetroxide staining [32] was performed with modification. Therefore, cells were washed four times in PBS, fixed (2% paraformaldehyde containing 0.1% glutaraldehyde, EM grade, Sigma-Aldrich, RT, 30 min) and osmium tetroxide-stained (0.1%, Sigma-Aldrich, RT, 30 min). The samples were washed three times in PBS, mounted in PBS and analyzed under bright field conditions.

### Total cholesterol measurement

Total lipid extractions from *E. bo*vis-infected (4, 8 and 17 days pi) and control BUVEC (*n* = 7) were performed in hexane:isopropanol according to Hara and Radin [33]. The cells were washed twice in ice-cold PBS, trypsinized and washed again (400 × *g*, 10 min). The total cell numbers were counted using a Neubauer chamber. Hexane:isopropanol (3:2, v/v) was added to the cell pellet. Cells were disrupted for 10 min in a Tissue Lyser (Qiagen, Hilden, Germany) using stainless steel beads. The samples were centrifuged (8000 × *g*, 1 min) and the supernatants were collected. The extraction was repeated once for each sample. The supernatants were then combined and dried manually under gentle liquid nitrogen stream. The total lipid extracts were reconstituted in 500 μL isopropanol:NP40 (9:1, v/v) followed by sonication in a waterbath (RT, 30 min). 5 μL of each sample were pre-treated with catalase [(5 μL of 0.5 mg/mL) in 40 μL of 1X reaction buffer (37 °C, 15 min)] in 96-well black clear-bottom plates (Greiner Bio-One) to reduce background fluorescence of peroxides in the solvents [34] before the enzyme cocktail of the Amplex-red kit (Life Technologies) was added. Cholesterol standards (Sigma-Aldrich; 10, 5, 2.5, 1.25, 0.625 and 0.325 μM) and blanks (solvent only) were included in each experiment. Fifty microliters of enzyme mixture (0.1 M potassium phosphate buffer, pH 7.4; 0.25 M NaCl, 5 mM cholic acid, 0.1% Triton X-100, 0.3 U/mL cholesterol oxidase, cholesterol esterase, 1.3 U/mL HRP, and 0.4 mM ADHP) were added and incubated (37 °C, 15 min). Resorufin formation was measured by fluorescence intensities (excitation wavelength of 530 nm, emission wavelength of 580 nm) in the Varioskan Flash Multimode Reader (Thermo Scientific). Total cholesterol of the samples was extrapolated to the values of the cholesterol standard. The total cholesterol content of each sample was normalized to its total cell number counts.

### Quantitative analysis of lipid droplet accumulation in infected BUVEC

*E. bovis*-infected and control BUVEC (*n* = 3) were trypsinized at days 8, 17 and 21 pi and pelleted in PBS (400 × *g*, 3 min, 4 °C). The resuspended cells were stained with Bodipy 493/503 (10 min, on ice) and washed twice with 1 ml PBS (400 × *g*, 3 min, 4 °C). The cells were transferred to 5-mL FACS tubes containing 200 μL of PBS and were processed in a FACS Calibur flow cytometer (BD Biosciences, Heidelberg, Germany) by laser excitation at 488 nm (FL1-H channel). Flow cytometry data were acquired by the BD CellQuest Pro software.

### Cholesterol, LDL and lipid droplet enrichment

For cholesterol enrichment, both, cholesterol and desmosterol (both Sigma-Aldrich) dissolved in ethanol were continuously added to BUVEC cultures (*n* = 5) at 5 μM final concentration. Although not being physiological the administration of cholesterol at low concentration (in ethanol) according to Xu et al. [35] was chosen to reduce its toxicity in long-term application. Supplementation of cholesterol complexed to cyclodextrin was not applicable to long-term culture in our hands owing to its high cytotoxicity.

For LDL enrichment, LDL (Sigma-Aldrich, 10 mg/mL final concentration) was supplemented to *E. bovis*-infected BUVEC (*n* = 5) from 10 days pi onwards. To artificially enhance lipid droplet formation in host cells, oleic acid (Sigma-Aldrich) was supplemented in BSA formulation complexes [36] to the cell culture medium. Direct conjugation was performed by mixing oleic acid-free BSA (fraction V, Roth) with oleic acid at the molar ratio of 6:1 (oleic acid:BSA). BUVEC (*n* = 3) were treated with 5 μM oleic acid/BSA complexes for an induction period of 1 h, and thereafter continuously with 2.5 μM to prevent toxicity. Treatments were repeated every second day from 8 days pi onwards.

### Low density lipoprotein (LDL) binding assay

The binding of LDL to receptors on the surface of infected cells and non-infected controls was estimated using Bodipy-labelled LDL (Life Technologies). Therefore, *E. bovis*-infected BUVEC (15 days pi) were cultivated in FCS-free endothelial cell basal medium (PromoCell) supplemented with 10% lipoprotein deficient-serum (Sigma-Aldrich) for 36–48 h prior to medium supplementation with Bodipy-labelled LDL (10 μg/mL). The cells were incubated for 1 h at 4 °C followed by 4 h at 37 °C and also 3 h, 5 h for fluorescence microscopy. For qualitative analyses the cells were washed in PBS, fixed (4% paraformaldehyde, 10 min) and mounted in Prolong antifading mounting medium prior to fluorescence microscopy. For quantitative analyses, BUVEC were treated with 3% NaN_3_ (5 min, RT) to inhibit receptor recycling and washed with ice-cold PBS. Monolayers were detached by Accutase (Sigma-Aldrich) treatment (5 min, 37 °C), washed in PBS (400 × *g*, 5 min) and transferred to 5-mL FACS tubes containing 200 μL of PBS. The cells were processed in a FACS Calibur flow cytometer using the FL1-H channel.

### Real-time quantitative PCR assay for the detection of cholesterol metabolism-associated molecules

Primers and probes (Biomers, Ulm, Germany) were designed using Beacon Designer 7 (Premier Biosoft) and Primer 3 (NCBI) software. In silico analyses were performed to access primers and probes specificity [37]. Amplification efficiency of each qPCR assay was determined using linearized plasmids containing the respective amplicon in pDrive (Qiagen). Only systems showing efficiency values >0.9 were used. The sequences of primers and probes and the efficiencies of each system are listed in Table 1.

**Table 1:** **Sequences of primers and probes used in real-time qPCR**

**Symbol**	**Name**	**Accession number**	**PCR efficiency**	**Amplicon length**	**Forward (5’→3’)**	**Reverse (5’→3’)**	**Probe (reporter 5’-3’ quencher)**
ACAT1	Acetyl-CoA acetyltransferase 1	NM_001046075.1	0.95	102	TCATATGGGCAACTGTGCTGA	CTGCTTTACTTCTGGTATAG	FAM-AGCATAAGTATCCTGTTCCTCTCGTG-BHQ1
ACAT2	Acetyl-CoA acetyltransferase 2	NM_001075549.1	1.08	194	AGCAGTGGTTCTTATGAA	AGGCTTCATTGATTTCAAA	FAM-ATCAACATCCTCCAGCGACCA-BHQ1
HMGCS1	3-hydroxy-3-methylglutaryl-CoA synthase 1	NM_001206578	0.93	197	CTACCTCAGTGCATTAGA	CTCTGTTCTGGTCATTAAG	HEX-AAGTCATTCAGCAACATCCGAGC-BHQ1
HMGCR	3-hydroxy-3-methylglutaryl-CoA reductase	NM_001105613.1	0.94	109	GCCATCAACTGGATAGAG	CCTCAATCATAGCCTCTG	FAM-TCTCTGACAACCTTGGCTGGAAT-BHQ1
SQLE	Squalene epoxidase	NM_001098061.1	0.93	132	CCCTTCTTCACCAGTAAA	CCCTTCAGCAATTTTCTC	HEX-AACAACAGTCATTCCTCCACCAGTA-BHQ1
CH25H	Cholesterol 25-hydroxylase	NM_001075243.1	1.00	87	TTGGGTGTCTTTGACATG	CAGCCAGATGTTGACAAC	FAM-CGTCTTGCTGCTCCAGTGTC-BHQ1
SOAT1	Sterol O-acyltransferase 1 (SOAT1)	NM_001034206.2	0.93	99	GCCCTCCTCATTCTCTTC	CCCAAAAGCATAAGACATGAG	FAM-AGCACCAGCCTTCCTTCATCA-BHQ1
LDLR	Low density lipoprotein receptor	NM_001166530.1	1.00	96	CGCCTACCTCTTCTTTAC	ACCACGTTCTTAAGGTTG	FAM-TCGCTTCGGTCCAGAGTCATC-BHQ1
OLR1	Oxidized low density lipoprotein/(lectin-like) receptor 1	NM_174132.2	0.97	102	CAAGCTGGATGAGAAATC	GGACAAGGACCTGAATAG	TET-ACTTCACCGCCAGAACCTGA-BHQ1
GAPDH	Glyceraldehyde-3-phosphate dehydrogenase	AF022183	0.98	82	GCGACACTCACTCTTCTACCTTCGA	TCGTACCAGGAAATGAGCTTGAC	FAM-CTGGCATTGCCCTCAACGACCACTT–BHQ1

*E. bovis*-infected and control BUVEC (*n* = 3) were processed for total RNA isolation at different time points of macromeront maturation (days 12, 14, 17 and 20 pi). Total RNA isolation was performed using the RNeasy kit (Qiagen) according to manufacturer’s protocol. Therefore BUVEC were lysed within the cell culture flasks with RLT lysis buffer (600 μL/25 cm^2^ flask) and processed as proposed by the manufacturer. An on-column DNase treatment (Qiagen) according to manufacturer’s instruction was included in the total RNA isolation procedure. Total RNAs were stored at −20 °C until further use. The quality of total RNA samples was controlled via Agilent 2100 bioanalyzer (Agilent Technologies, USA). In order to remove any DNA leftover, a second genomic DNA digestion step was performed. Therefore, 1 μg of total RNA was treated with 10 U DNase I in 1x DNase reaction buffer (all Thermo Scientific, 37 °C, 1 h). DNase was inactivated by heating the sample (65 °C, 10 min). The efficiency of genomic DNA digestion was controlled by including no-RT-controls in each real-time PCR experiment.

cDNA synthesis was performed using the SuperScript III First-Strand Synthesis System according to manufacturer’s protocol with slight modifications.1 μg of DNase-treated total RNA was added to 1 μL of 50 μM oligo d(T), 1 μL of 50 ng/μL random hexamer primer, 1 μL of 10 mM dNTP mix and DEPC-treated water was adjusted to 10 μL total volume. The sample was incubated at 65 °C for 5 min and then immediately cooled on ice. For first strand cDNA synthesis, 2 μL of 10x RT buffer, 1 μL 25 mM MgCl_2,_ 1 μL 0.1 M DTT, 1 μL RNaseOUT (40 U/μL) and 1 μL SuperScript III enzyme (200 U/μL) were added and the sample was incubated at 50 °C for 60 min followed by a 85 °C-inactivation step for 15 min.

Real-time PCR was performed in duplicates in a 10 μL total volume containing 400 nM forward and reverse primers, 200 nM probe, 10 ng cDNA and 5 μL 2× PerfeCTa qPCR FastMix (Quanta Biosciences, USA). The reaction conditions for all systems were as follows: 95 °C for 10 min, 40 cycles at 95 °C for 10 s, 60 °C for 15 s and 72 °C for 30 s. PCRs were performed on a Rotor-GeneQ cycler (Qiagen, Hilden, Germany). No-template controls (NTC) and no-RT reactions were included in each experiment. As a reference gene we chose with GAPDH a gene that was previously used in other publications reporting on *E. bovis*-infected BUVEC [24,38]. To prove that the GAPDH gene is a suitable reference gene in this experimental set-up, we have previously performed experiments on the stability of GAPDH gene transcription in *E. bovis*-infected BUVEC. Therefore, four different BUVEC isolates (=4 biological replicates) were infected with *E. bovis* and tested for GAPDH gene transcription (in duplicates) throughout infection (at days 4, 8, 12, 14 and 17 pi). Considering all samples of all isolates at C_t_ level we showed that GAPDH gene transcription was not significantly influenced by the infection (mean ± standard deviation of the C_t_s of all samples: 21.31 ± 0.84) but proved stabile within the kinetics, since the variation of the data accounted to less than 4%. Analyses of the qPCR data used the comparative C_t_ method (ΔΔ*C*_T_ method) and reported as n-fold differences comparing *E. bovis*-infected BUVEC with non-infected ones after normalizing the samples with the GAPDH reference gene.

### Merozoites I quantification via *Eimeria bovis* microneme protein 4 (EbMIC4)-based real-time PCR

For merozoites I quantification we used a real-timePCR system based on the detection of the single copy gene of *E. bovis* microneme protein 4 (EbMIC4, [39]) applying the following primers and probe: forward primer: 5’-CACAGAAAGCAAAAGACA-3’, reverse primer: 5’-GACCATTCTCCAAATTCC-3’ and probe: 5’-FAM-CGCAGTCAGTCTTCTCCTTCC-BHQ1-3’ [26]. Therefore, cell culture supernatants containing merozoites I were collected at indicated time points pi. At the end of the culture period the cells were trypsinized and collected together with the supernatant followed by centrifugation (600 × *g*, 15 min). The cell pellet was treated with 200 μL of cell lysis buffer containing 0.32 M Sucrose, 1% Triton X-100, 0.01 M Tris-HCl (pH 7.5), 5 mM MgCl_2_ and incubated in 100 μL 1X PCR buffer (Quanta, USA) containing 20 μL proteinase K (20 mg/mL; Qiagen) at 56 °C for 1 h. Proteinase K was heat-inactivated (95 °C, 10 min) and the DNA-containing samples were frozen at − 20 °C until further use. All samples were processed as triplicates.

Real-time PCR was performed in a total volume of 20 μL containing 5 μl DNA of merozoites I samples, 400 nM of each primer, 200 nM probe and 10 μL PerfeCTa MasterMix (Quanta, USA) at 95 °C for 10 min; 40 cycles at 95 °C for 10 s, 60 °C for 15 s and 72 °C for 30 s. In each PCR experiment serial dilutions of merozoites I covering 6 magnitude orders of 10-fold (10^6^-10) were included allowing for absolute quantification of merozoites I counts in the samples.

### Statistics

Real-time PCR data analysis was performed by the ΔΔ*C*_T_ method, normalized to GAPDH and expressed as relative fold change compared to control [40,41]. For statistics data were analyzed by Student’s *t*-tests comparing control and treated groups. GraphPad Prism 6.02 was used to visualize graphs.

## Results

### Free cholesterol and neutral lipid/lipid droplet distribution in *E. bovis* invasive stages

**Figure 1:**
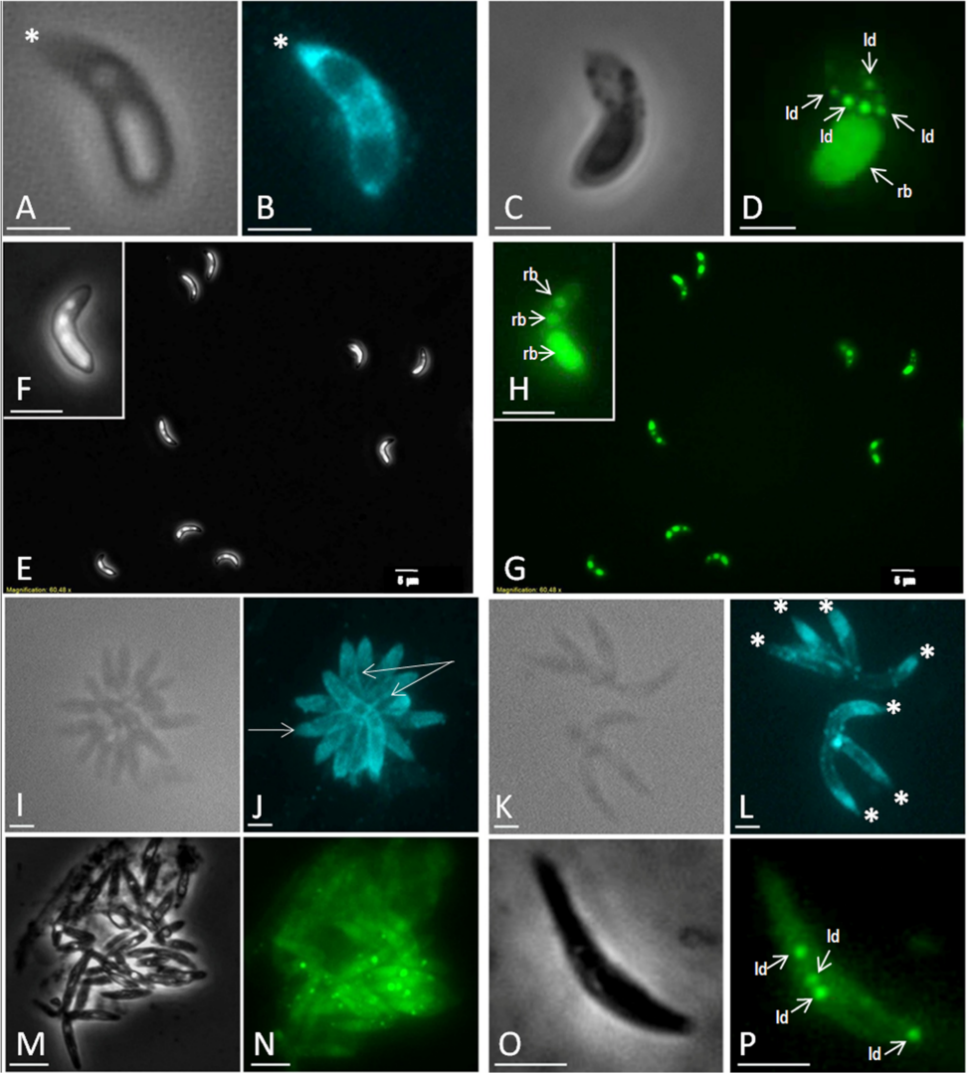
**Cholesterol and neutral lipid/lipid droplet localization in free** ***E. bovis*** **stages.** Freshly excysted *E. bovis* sporozoites were stained by filipin (**A** = phase contrast) and Bodipy 493/405 (**C** = phase contrast). Note the bright filipin signal in the apical part (*) and pellicula (**B**) and the strong Bodipy 493/405 staining of the refractile bodies (rb) and cytosolic lipid droplets (ld) (**D**). Refractile bodies but no lipid droplets were stained in sporozoites by Nile red (**G**, **H** = close-up, **E**/**F** = phase contrast). Merozoites I showed strong filipin-induced signals in the pellicula (**J** arrows, **I** = phase contrast) and at the apical tips (**L** *, **K** = phase contrast). Bodipy 493/405 staining revealed cytosolic lipid droplets in merozoites I (**N**, **P** arrows; **M**, **O** = phase contrast). Scale bars represent 5 μm.

*E. bovis* sporozoites and merozoites I both were intensively stained by filipin (Figures 1B, J, L). The strongest reactions were detected at the apical part and in the plasma membrane of these stages indicating high free cholesterol contents in the apical complex and the pellicula (Figures 1B, J, L). In addition, an intense staining was observed in the cytoplasm of sporozoites (Figure 1B). These reactions may origin from intraparasitic organelle membranes. Sporozoites and merozoites I of *E. bovis* also showed an intense staining by Nile red (Figures 1G and H) and Bodipy 493/503 (Figures 1D, N, P), indicating a high content of neutral lipids/lipid droplets within these stages. However, using Nile red we failed to demonstrate structurally defined lipid droplet-like structures within the cytoplasm of these stages, whilst Bodipy 493/503 staining resulted in strong fluorescent globular lipid body-like structures (Figures 1D and P). In merozoites I and sporozoites lipid droplets were situated in the cytoplasm and differed in numbers per specimen. Overall, up to 8 lipid droplet-like structures were detected per invasive stage. The overall strongest reactions upon Nile red or Bodipy 493/503 staining were found in the refractile bodies of the sporozoite stages (Figures 1D, G, H, merozoites I do not contain any refractile bodies) indicating the storage of neutral lipids within these specific apicomplexan organelles.

### Cholesterol and lipid droplets accumulate in *E. bovis*-infected cells during macromeront formation

**Figure 2:**
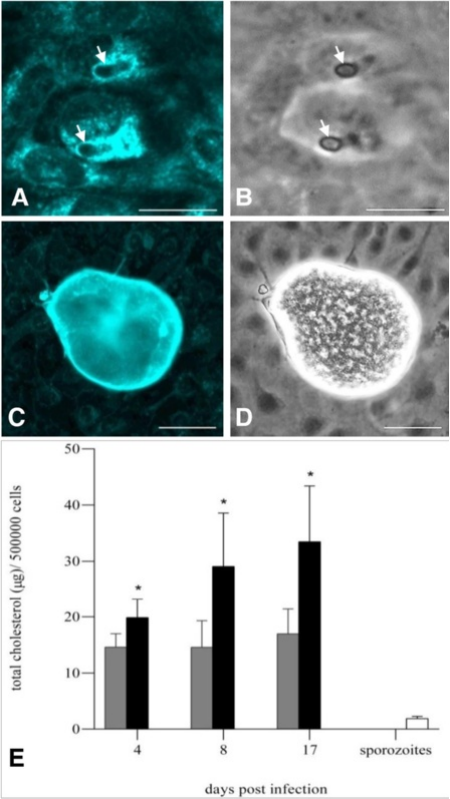
**Cholesterol distribution and cholesterol content in** ***E. bovis*** **infected host endothelial cells.** Bovine umbilical vein endothelial cells (BUVEC) were infected with *E. bovis* sporozoites and stained with filipin at one day (**A**; **B** = phase contrast, arrows indicate sporozoites) and 17days (**C**; **D** = phase contrast) pi. Note the bright filipin staining adjacent to intracellular sporozoites (**A**) and the strong reactions in a meront I-carrying cell (**C**) compared to non-infected cells in the neighborhood. For the quantification of the total cholesterol contents (**E**), infected (black bars) and non-infected (grey bars) BUVEC (*n* = 7) were harvested at different time points after infection (4, 8, 17 days pi), processed for total lipid extraction and total cholesterol measurement using the Amplex Red® kit. Pure sporozoites (processed numbers correspond to the infection dose) were used as controls. Scale bars represent 10 μm.

One day after host cell infection, filipin-positive signals accumulated very close to the parasites intracellular position within the PV (Figure 2A). Compared to non-infected control cells directly adjacent to infected host cells, the cholesterol abundance was also significantly enhanced in immature and mature meronts I (Figure 2C). Owing to the massive enlargement of the host cell leading to a close position of the PV and host cell membrane, it cannot be concluded whether the signals originate merely from the parasite and its PVM or from the host cell membrane or both. Total cholesterol quantification in lipid extracts revealed a significant increase of cholesterol abundance in *E. bovis*-infected host cells over time when compared to non-infected controls (Figure 2E; 4, 8 and 17 days pi vs. controls: all *p* < 0.01). Given that the cholesterol content of sporozoites (here the individual infection dose of 5 × 10^5^ sporozoites was analyzed, Figure 2E) was rather low, the changes of host cellular cholesterol content could not be attributed to invading stages but to infection-triggered alteration of the cholesterol metabolism.

**Figure 3:**
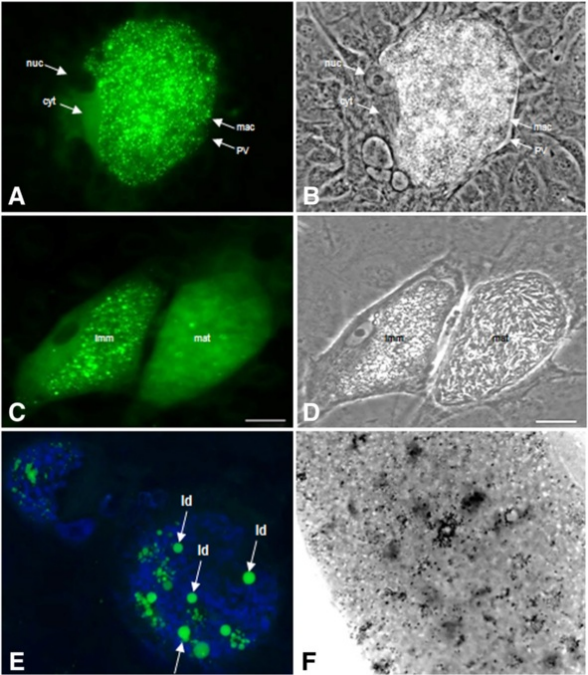
**Lipid droplet distribution in developing meronts.** Bovine umbilical vein endothelial cells (BUVEC) were infected with *E. bovis* sporozoites. For the illustration of lipid droplets, the cells (17 days pi) were either stained with Bodipy 493/503 and analyzed by conventional fluorescence microscopy (**A** + **C**: Bodipy 493/503 (green), **B** + **D**: respective phase contrast illustrations) or confocal microscopy (**E**: Bodipy 493/503 (green), DNA staining via DAPI (blue); illustration in (**E**) was projected from 25 slices of Z-stack confocal analysis which is given in Additional file 1) or subjected to osmium tetroxide staining (**D** including close-up). Note massive abundance of lipid droplets (green (Bodipy staining) or black (osmium tetroxide) dots) within immature macromeronts (**A**, **C**, **F**) and the large sizes of lipid droplets (= ld, **E**). Note also that the cytoplasm of infected host cells is free of lipid droplets (**A**, **C**). Scale bars represent 20 μm. nuc = nucleus of the host cell, cyt = cytoplasm of the host cell, PV = parasitophorous vacuole, mac = macromeront-derived plasma membrane, imm = immature macromeront, mat = mature macromeront, ld = lipid droplet.

Based on Bodipy 493/503 staining, a considerable enhancement of lipid droplet formation was observed with ongoing macromeront development (Figure 3). The most significant accumulation of distinct lipid droplet-like structures occurred in immature macromeronts (15-17 days pi, Figures 3A and 3C). The Bodipy 493/503 staining was cross-checked with Nile red staining (data not shown) which showed the same feature. Alongside with macromeront maturation and merozoite I formation, the fluorescence pattern changed from a spotty appearance illustrating single lipid droplets to a rather cloudy and diffuse reaction indicating that lipid droplet contents were almost totally exhausted or consumed for merozoite I formation (Figure 3C). In order to better define lipid droplet position, structure and size within *E. bovis*-infected cells, confocal microscopic analyses were also applied for Bodipy 493/503-stained samples. Detailed analyses of *E. bovis*-infected host cells 17 days pi revealed the presence of bright fluorescent lipid droplets throughout the macromeront corpus as demonstrated by the detection in each layer (Z-stack) of the specimens (single layer: Figure 3E, all layers see Additional file 1). Lipid droplets showed classical globular shapes but were of differing sizes. Whilst most lipid droplets were rather small (<1 μm), some of them revealed a size of more than 5 μm in diameter.

Given that the fixation mode may alter lipid droplet integrity leading to dimmed LD appearance [42], we additionally used osmium tetroxide staining which is known to better preserve lipid body structures. However, osmium tetroxide treatments led to similar results to those of Bodipy 493/503 staining thus confirming the significant increase of lipid droplet abundance (visible as small black dots) in *E. bovis* macromeront-carrying host cells (Figure 3F).

**Figure 4:**
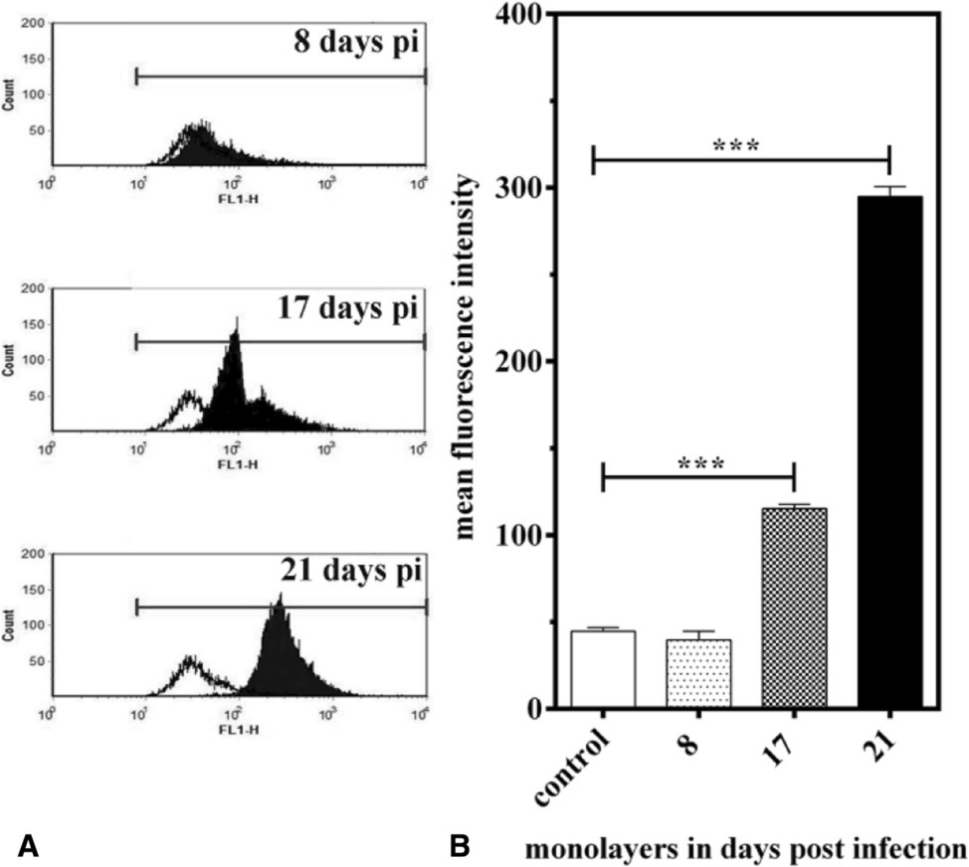
**Lipid droplet abundance in** ***E. bovis*** **-infected endothelial host cells.** Bovine umbilical vein endothelial cells (BUVEC, *n* = 3) were infected with *E. bovis* sporozoites. The cells were stained with Bodipy 493/503 to trace lipid droplets and subjected to flow cytometry analyses at 8, 17 and 21 days pi. Infected cells were assigned according to their size and granularity. **A** exemplary illustrations of histograms of Bodipy 493/503-positive infected (black histogram) and non-infected (white histogram) BUVEC on days 8, 17 and 21 pi; (**B**) mean and standard deviations of lipid droplet contents in infected host cells at 0, 8, 17 and 21 days pi.

Applying FACS technology we additionally quantified the changes of total lipid droplet contents in infected BUVEC and control cells. FACS analyses revealed a significant increase (2.8- and 6.6-fold at 17 and 21 days pi, respectively) of total lipid droplet abundance of *E. bovis*-infected BUVEC in times of macromeront formation resulting in highly significant values for days 17 and 21 pi compared to non-infected controls (both *p* ≤ 0.0001, Figure 4). These data confirmed the microscopic observations described above and underlined the relevance of lipid droplet formation during *E. bovis* macromeront development.

### Extracellular sterol enrichment and enhanced cellular lipid droplet disposability boost parasite replication

**Figure 5:**
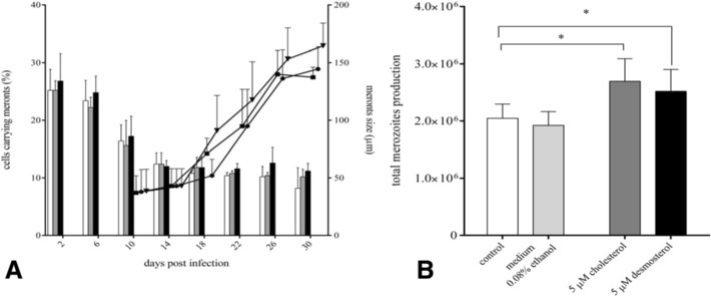
***E. bovis*** **macromeront formation and merozoiteI production after cholesterol and desmosterol supplementation.** *E. bovis*-infected BUVEC (*n* = 5) were cultivated in non-supplemented (=controls) or cholesterol- and desmosterol-enriched medium. The effects on meront I formation (**A**) were assessed microscopically by estimating the rate of meront I-carrying host cells (black bars: cholesterol-enriched medium, grey bars: desmosterol-enriched medium, white bars: controls) and by measuring the sizes of developing meronts I (triangles: cholesterol-enriched medium, circles: desmosterol-enriched medium, squares: controls). The effects on total merozoite Iproduction (**B**) were quantified via an EbMIC4-based qPCR after 30 days pi.

To assess the effects of exogenously supplied cholesterol on *E. bovis* macromeront development, free sterols (cholesterol and desmosterol) were administered to cell cultures. Desmosterol is a cholesterol intermediate precursor and can replace cholesterol function in sustaining cell proliferation [43]. Owing to long term cytotoxicity we could not supply cholesterol in the cyclodextrin-complexed form and chose the very simple approach of supplementation in ethanol which had previously been shown to work in *T. gondii* cultures [4] and according to Xu et al. [35]. To avoid intracellular crystallization and cytotoxicity [35], low concentrations (5 μM) of these substituents were applied. We observed a significant beneficial effect on merozoite I production leading to enhanced offspring generation in both, cholesterol- and desmosterol-treated host cells when compared to non-treated *E. bovis*-infected cultures (Figure 5B, *p* ≤ 0.05). However, the infection rates and macromeront sizes were not significantly altered by cholesterol or desmosterol supplementation (Figure 5A).

**Figure 6:**
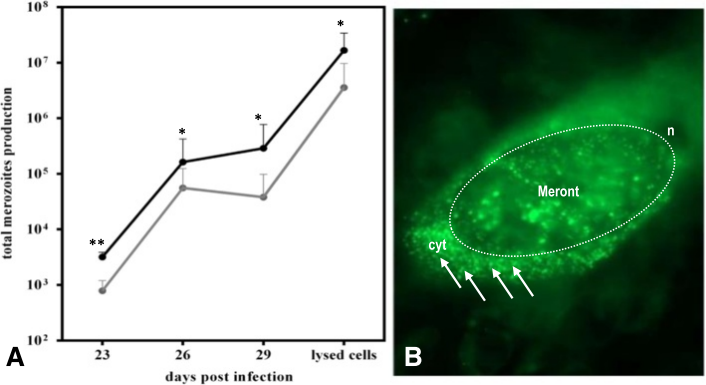
***E. bovis*** **merozoite I production after artificial lipid droplet induction via oleic acid supplementation.** *E. bovis*-infected BUVEC (*n* = 3) were cultivated in non-enriched (=controls, grey circles, **A**) or oleic acid-enriched (black circles, **A**) medium. At different time points after infection (23, 26, 29 and 30 days pi) the numbers of merozoites I present in cell culture supernatants (**A**) were quantified via an EbMIC4-based qPCR. **B** Exemplary illustration of a meront I- (indicated by a dotted circle) carrying, infected host cell (17 days p. i.) being cultivated in oleic acid-enriched medium. Note the massive increase of lipid droplets in the cytoplasm of the host cell (cyt) and in the meront. n = nucleus of the host cell.

Given that lipid droplets may play a pivotal role in *E. bovis* in vitro development, we assessed the effects of an artificially enhanced abundance of lipid droplets in BUVEC. Therefore, BUVEC were treated with oleic acid, a well-known inducer of lipid droplets formation in several types of mammalian cells [36]. The microscopic control of oleic acid-treated, infected BUVEC confirmed the lipid droplet-boosting function of oleic acid treatments since many more lipid droplets were found within the cytoplasm of treated cells when compared to non-treated ones (Figure 6B, compare to Figures 3A and 3C). Kinetic analyses of merozoite I in vitro production in oleic acid-treated and non-treated infected cells confirmed the key role of lipid droplets in optimal parasite proliferation. Thus, significant beneficial effects of oleic acid treatments on merozoite I production were observed at all time points of merozoite I quantification (Figure 6A; *p* ≤ 0.01 for days 23; *p* ≤ 0.05 for days 26, 29, 30 pi). Referring to the total merozoite I production, a 4.7 ± 2.9 fold increase of offspring production was estimated.

### *E. bovis* infection enhances surface LDL receptor abundance on infected cells and exogenously supplied LDL improves merozoite I production

**Figure 7:**
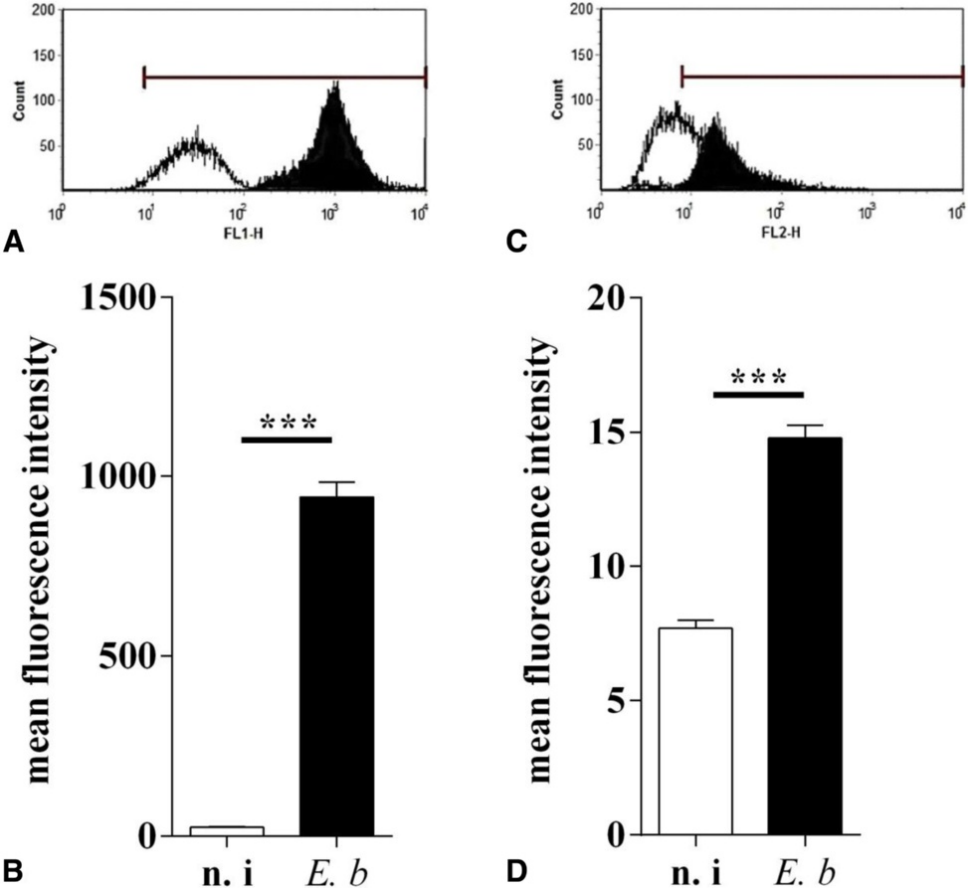
**LDL-binding and LDL surface receptor abundance on** ***E. bovis*** **-infected endothelial host cells. A**-**B**: *E. bovis*-infected BUVEC and non-infected controls were cultured in LDL-depleted medium for 36-48 h then incubated in medium containing Bodipy-labelled LDL. After incubation at 4 °C for 1 h followed by 4 h of incubation at 37 °C, LDL binding was measured by FACS analysis: exemplary histogram (**A**) and LDL quantification in infected (black bar, 17 days pi) and non-infected (white bar) cell layers. **C**-**D**: For the quantification of surface LDL receptors, infected (black bar 17 days pi) and non-infected (white bar) cell layers were reacted with LDL receptor (LDLR)-specific primary antibodies and appropriate FITC-conjugated secondary antibodies and subjected to flow cytometry analyses: exemplary histogram (**C**) and LDLR expression on the surface of *E. bovis*-infected (black bar, 17 days pi) and non-infected (white bar) BUVEC.

To assess whether LDL binding to the cell surface is altered in infected host cells, Bodipy-labelled LDL was supplemented to in vitro cultures that had previously been starved in LDL-free medium. LDL binding was quantified using FACS technology. A significant increase of Bodipy-LDL binding was detected in infected cells versus non-infected controls (*p* < 0.0001, Figures 7A and 7B). Overall, a 37.5-fold enhancement was measured in *E. bovis*-infected cells.

To estimate whether the increase in LDL-binding originated from enhanced surface LDL receptor (LDLR) abundance, a flow cytometry-based assay using an LDLR-specific antibody was established and tested on *E. bovis*-infected BUVEC at 17 days pi. Overall, the data revealed a significant infection-induced increase of LDLR surface expression (1.92 fold) in macromeront-carrying host cells (infected vs. non-infected cells: *p* < 0.0001; Figures 7C and 7D). These data are in agreement with transcriptional profiles of LDLR in *E. bovis*-infected cells, as described later (Figure 9).

**Figure 8:**
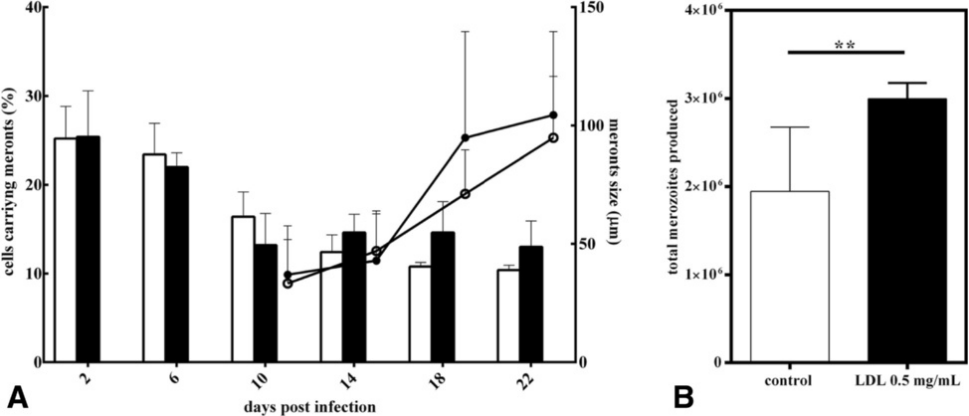
***E. bovis*** **macromeront formation and merozoite I production after LDL supplementation.** *E. bovis*-infected BUVEC (*n* = 5) were grown in non-enriched (=controls) or LDL-enriched medium. The effects of LDL supplementation on macromeront formation (**A**) were assessed microscopically by estimating the rate of macromeront-carrying host cells (black bars: LDL-enriched medium, white bars: controls) and by measuring the size of developing macromeronts (black circles: LDL-enriched medium; white circles: controls). The effects of LDL supplementation on total merozoite I production (**B**) were quantified via a EbMIC4-based qPCR at 30 days pi.

Given that LDL binding and LDLR surface expression is enhanced in *E. bovis*-infected host cells, we then investigated whether exogenous LDL supplementation would be of benefit for *E. bovis* macromeront development in vitro. Indeed, excess LDL stimulated *E. bovis* merozoite I production. Although, macromeront sizes and rates increased only slightly (Figure 8A), we observed a significant increase of the total merozoite I production after LDL supplementation (*p* < 0.01, Figure 8B) resulting in a 1.54-fold enhancement of offspring synthesis.

### *E. bovis* infection induces up-regulation of the gene transcription of molecules involved in the mevalonate pathway and in cholesterol hydroxylation and esterification

**Figure 9:**
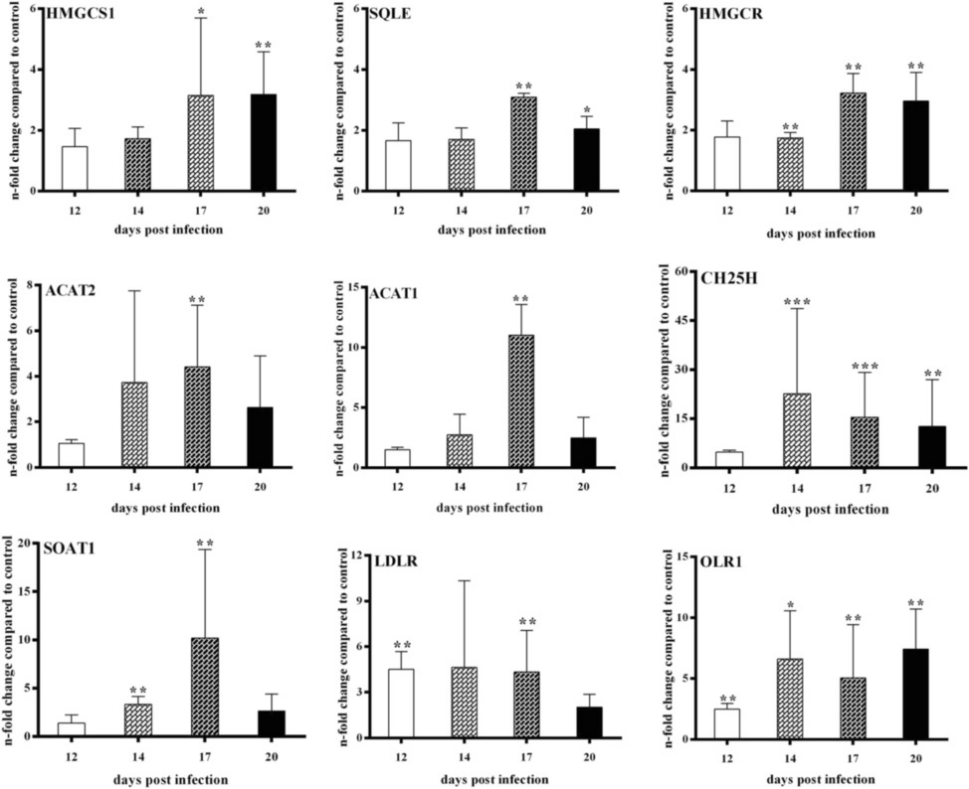
**Gene transcription of molecules involved in cholesterol** ***de novo*** **synthesis and cholesterol esterification and hydroxylation in** ***E. bovis*** **-infected endothelial host cells.** Total RNA was isolated from *E. bovis*-infected BUVEC (*n* = 3) at different time points of macromeront formation (12, 14, 17 and 20 days pi), reverse transcribed and subjected to quantitative real-time PCR assays for the detection of bovine acetyl-CoA acyl transferase 1 (ACAT1), acetyl-CoA acyl transferase 2 (ACAT2), HMG-CoA synthase 1 (HMGCS1), HMG-CoA reductase (HMGCR), squalene epoxidase (SQLE), sterol O-acyltransferase 1 (SOAT1), cholesterol 25-hydroxylase (CH25H), low density lipoprotein receptor (LDLR) and oxidized low density lipoprotein receptor 1 (OLR1). Non-infected BUVEC were equally processed at each time point of the experiments and served as negative controls.* = *p* ≤ 0.05, ** = *p* ≤ 0.01, *** = *p* ≤ 0.0001.

Host cell de novo biosynthesis of cholesterol is a multistep metabolic pathway involving more than 30 enzymatic reactions. In these experiments transcriptional profiles of several relevant molecules were analyzed during *E. bovis* macromeront formation in vitro. For the formation of acetoacetyl-CoA representing the substrate of the mevalonate pathway, ACAT1/ACAT2 activities are needed [44]. Gene transcription profiles of *E. bovis*-infected BUVEC during macromeront formation revealed the highest and significant up-regulation for both molecules at 17 days pi (*p* ≤ 0.01) indicating a high demand of acetoacetyl-CoA when merozoites I are to be formed (Figure 9). Overall, the up-regulation of ACAT1 gene transcription (maximum: 11.03 ± 2.53-fold) was higher than that of ACAT2 (maximum: 4.41 ± 2.1-fold). In addition, the gene transcriptions of HMGCS1, HMGCR and SQLE were all up-regulated during *E. bovis* macromeront formation with significant reactions at 17 and 20 days pi (Figure 9, HMGCS1: 17 days pi: *p* ≤ 0.05, 20 days pi *p* ≤ 0.01; HMGCR: 14, 17 and 20 days pi: *p* ≤ 0.01; SQLE: 17 days pi: *p* ≤ 0.01, 20 days pi: *p* ≤ 0.05). These data explicitly indicate that *E. bovis* macromeront development significantly interferes with the host cell de novo biosynthesis via the mevalonate pathway. Given that all molecules were equally found up-regulated, these reactions may reflect the strong need of the parasite for excess cholesterol synthesis.

Following de novo biosynthesis, cholesterol may either be recycled to the membranes or modified via further enzymatic steps promoting hydroxylation via CH25H or esterification via SOAT1 [45–47]. Analyses on *E. bovis*-infected BUVEC revealed that both pathways of cholesterol processing were significantly up-regulated at times of macromeront formation. Thus, SOAT1 gene transcripts were found significantly increased for 3.3- and 10.2-fold at 14 and 17 days pi (both: *p* ≤ 0.01), respectively, when compared to non-infected controls (Figure 9). Given that cholesterylesters are stored in lipid droplets, these results indirectly confirmed the data on enhanced lipid droplet formation in *E. bovis*-infected host cells. The overall strongest up-regulation of all gene transcripts tested was measured for CH25H (Figure 9). Hence, significant enhancements of gene transcription were detected at days 14, 17 (both: *p* ≤ 0.001) and 20 (*p* ≤ 0.01) pi, suggesting a crucial role of 25-hydroxycholesterol synthesis in the development of *E. bovis* macromeronts.

The transcriptional profiling of LDLR and OLR1 in *E. bovis*-infected host cells additionally showed significantly enhanced mRNA levels for both molecules. Compared to non-infected controls, significant reactions were detected for LDLR at 12 and 17 days pi (both: *p* ≤ 0.01) reaching an up to 9.49-fold increase (Figure 9). OLR1 gene transcripts were significantly enhanced during the entire period of investigation (days 12, 17 and 20 pi: *p* ≤ 0.01, day 14 pi: *p* ≤ 0.05, Figure 9) reaching an average of up to 7-fold increase in *E. bovis* infected cells.

## Discussion

During first merogony *E. bovis* forms the PV, enlarges the endothelial host cell and produces thousands of merozoites I and, consequently, is in considerable need for cholesterol. We here show that the parasite significantly utilizes different host cellular cholesterol acquisition pathways and demonstrate that *E. bovis*-induced modulation of the host cell cholesterol metabolism differs from other apicomplexan parasites for several reasons: i) the massive induction of lipid droplets within the immature meront I stage, ii) the simultaneous interference with both, the host cellular de novo synthesis and LDL-mediated cholesterol up-take, iii) the involvement of OLR1 and iv) the induction of CH25H in host cellular cholesterol modification.

In contrast to other apicomplexan parasites, such as *T. gondii*, the monoxenous protozoa *E. bovis* represents a strict host- and host cell-specific parasite. Thus, first merogony exclusively occurs in bovine endothelial cells and lasts for up to three weeks. Filipin staining experiments ascertained that *E. bovis* stages indeed do contain free cholesterol molecules. This is in line with tachyzoites of *T. gondii*, [4,47–49] but in contrast to *P. yoelii* free sporozoites which lack filipin staining [6]. Early after infection and in cells carrying developing macromeronts strong filipin reaction were observed indicating high cholesterol contents in infected cells. Quantitative analyses revealed an almost doubling of total cholesterol content in times of *E. bovis* macromeront maturation. Furthermore and in accordance to studies on *T. gondii* [4], excess exogenous cholesterol resulted in significantly enhanced offspring production confirming the key role of high cholesterol abundance for successful *E. bovis* development.

Neutral lipid staining in *E. bovis* free stages revealed a differential distribution of free cholesterol and neutral lipids in sporozoites and merozoites I. Thus, neutral lipids exclusively occurred in the refractile bodies of sporozoites and in cytosolic lipid droplet-like structures of both stages. This is in line with observations on *E. tenella* sporozoites [50,51] and *T. gondii* tachyzoites [4,12,15].

The most striking feature in *E. bovis*-infected host cells was the massive induction of lipid droplets within the immature meront I (note that the cytoplasm did not show any lipid droplets) which finds no correlate in any other coccidian infection. Although a stage-dependent increase of lipid droplets was also reported in *P. falciparum*-infected erythrocytes [19–21], these organelles were mainly localized in the host cell cytoplasm and not within the parasitic stages and were of much less numbers. Interestingly, the maximum lipid droplet generation was observed in late immature macromeronts, i.e. at times when merozoites I are about to be formed. In contrast, in mature macromeronts carrying fully developed merozoites I, structurally defined lipid droplets were hardly observed, suggesting that lipid droplet contents were almost totally consumed during offspring formation. Confocal microscopy analyses revealed considerable variations of lipid droplet sizes in macromeront-carrying host cells, ranging from submicrometer diameters to large sizes of ≥5 μm. Accordingly, “gigantic” lipid droplets (being situated within the host cell cytoplasm) were previously described in *Trypanosomacruzi*-infected host cells [52] but not in any other coccidian parasite, so far. The general phenomenon of enhanced lipid droplet formation in infected host cells was also reported for other protozoan parasites such as *T. cruzi, T. gondii*, *P. berghei* or *P. falciparum* [16,18,19,52–54]. However, it has to be stressed that the localization and the degree of lipid droplet formation in the latter cases was not at all comparable to *E. bovis* infections and most reports referred to host cell cytosolic lipid droplets and not parasitic ones. Whilst in *T. cruzi*-, *T. gondii-* or *P. falciparum*-induced host cell lipid droplets never exceeded a number of ~20 [16,19,55], the numbers of these organelles was 100-1000-fold higher in immature *E. bovis* meronts (suspected numbers since the true numbers are uncountable owing to the thickness of the specimens). The key role of lipid droplets was furthermore proven by oleic acid treatments which significantly improved parasite proliferative capacities. Given that excess cellular cholesterol is stored in the esterified form in lipid droplets, we additionally analyzed the gene transcription of SOAT1, i.e., the enzyme promoting cholesterol esterification, and demonstrated a significant increase of respective transcripts in *E. bovis*-infected cells. In line with other apicomplexan parasites [14,15] the key role of cholesterol esterification for optimal *E. bovis* proliferation cells was recently confirmed by chemical SOAT inhibition leading to almost total blockage of *E. bovis* replication in endothelial host cells [37].

Besides esterification, hydroxylation of cholesterol is also performed to detoxify this molecule in mammalian cells. Transcriptional profiling and protein expression (P. Hamid, personal communication) analyses of *E. bovis*-infected BUVEC showed a significant up-regulation of CH25H which promotes cholesterol hydroxylation. It is noteworthy, that the overall highest up-regulation of gene transcripts and proteins concerned CH25H suggesting enhanced 25-OH-cholesterol (25-OHC) synthesis to occur in *E. bovis*-infected host cells. So far, no comparable phenomenon was reported in any other coccidian parasite infection. It has to be mentioned that levels of gene transcription and protein expression do not necessarily reflect cellular enzyme activities. However, preliminary results on biochemical 25-OHC measurements showed enhanced concentrations of this molecule in *E. bovis*-infected host cells (A. Taubert, unpublished data) indicating a new strategy of cholesterol processing in coccidian-infected host cells.

Transcriptional profiling of *E. bovis*-infected host cells showed that all molecules tested being involved in the mevalonate pathway or in early substrate synthesis were found up-regulated in times of merozoite I formation. An enhancement of ACAT1 was also confirmed on the protein level (P. Hamid, personal communication). These data are in line with recent data on chemical HMGCS1 and SQLE inhibition leading to a significant blockage of *E. bovis* proliferation [26]. Related data in *T. gondii* infections are somewhat conflicting: whilst Coppens et al. [4] denied any involvement of this pathway in *T. gondii*-infected host cells, other studies indicated that de novo synthesis indeed contributes to *T. gondii* growth [10,11,56].

Besides *de novo* biosynthesis, exogenous cholesterol is acquired via cellular lipoprotein internalization. LDL has been reported as major exogenous cholesterol source for *T. gondii*, *C. parvum* and *Plasmodium* spp. [4,6,7]. Accordingly, we here report on enhanced binding and up-take of LDL at times of *E. bovis* macromeront formation. Consistent with recent microarray data [25], LDLR gene transcripts were found significantly up-regulated throughout *E. bovis* macromeront development. Furthermore, a significant increase of surface LDLR abundance was detected on infected host cells via FACS analyses. These results are in line with data on *T. gondii*-infected CHO cells [4], but contrast to the fact that reduced LDLR expression did not affect the liver stage burden in the case of *Plasmodium* spp. [6]. In accordance to *T. gondii*-infected CHO cells [4], LDL supplementation had beneficial effects on *E. bovis* merozoite I production confirming the pivotal role of LDL for optimal parasite replication. In contrast, LDL enrichment had no stimulatory effects on *Plasmodium* spp. and *C. parvum* proliferation [6,7] or on *T. gondii* growth in macrophages [10] indicating parasite- and cell type-specific mechanisms.

The current transcriptional data additionally revealed the scavenger receptor OLR1 (syn. LOX-1) to be involved in *E. bovis*-triggered LDL-uptake since a strong up-regulation of this receptor was detected in times of macromeront formation. These data are in agreement with recent microarray data [25] and were confirmed on protein level (P. Hamid, personal observation.). OLR1 is considered as the major receptor for oxidized LDL (oxLDL) internalization in vascular endothelial cells [57–59]. To our knowledge, this represents the first report indicating an involvement of OLR1/oxLDL in coccidian host cell infections.

Overall, the current data indicate that *E. bovis* has the capacity to scavenge cholesterol from several cellular sources at a time, which clearly contrasts to *T. gondii* [4]. Given that cellular cholesterol synthesis is tightly regulated by a complex network of cellular mechanisms, more research is needed to understand how *E. bovis* outwits this regulatory network.

## Additional file

Additional file 1:
**Confocal sections of lipid droplet accumulation in a macromeront.** 25 confocal sections of the composite Z-stack shown in Figure 3E are depicted. Nuclei were stained by DAPI (blue) whilst lipid droplets were shown in green by Bodipy 493/503 staining. Scale bars represent: 10 μm.
