# Supplementary figures and images for: Eimeria bovis infection modulates endothelial host cell cholesterol metabolism for successful replication
Source: Vet Res. 2015 Sep 23;46(1):100. doi: 10.1186/s13567-015-0230-z (PMC4579583; doi:10.1186/s13567-015-0230-z)

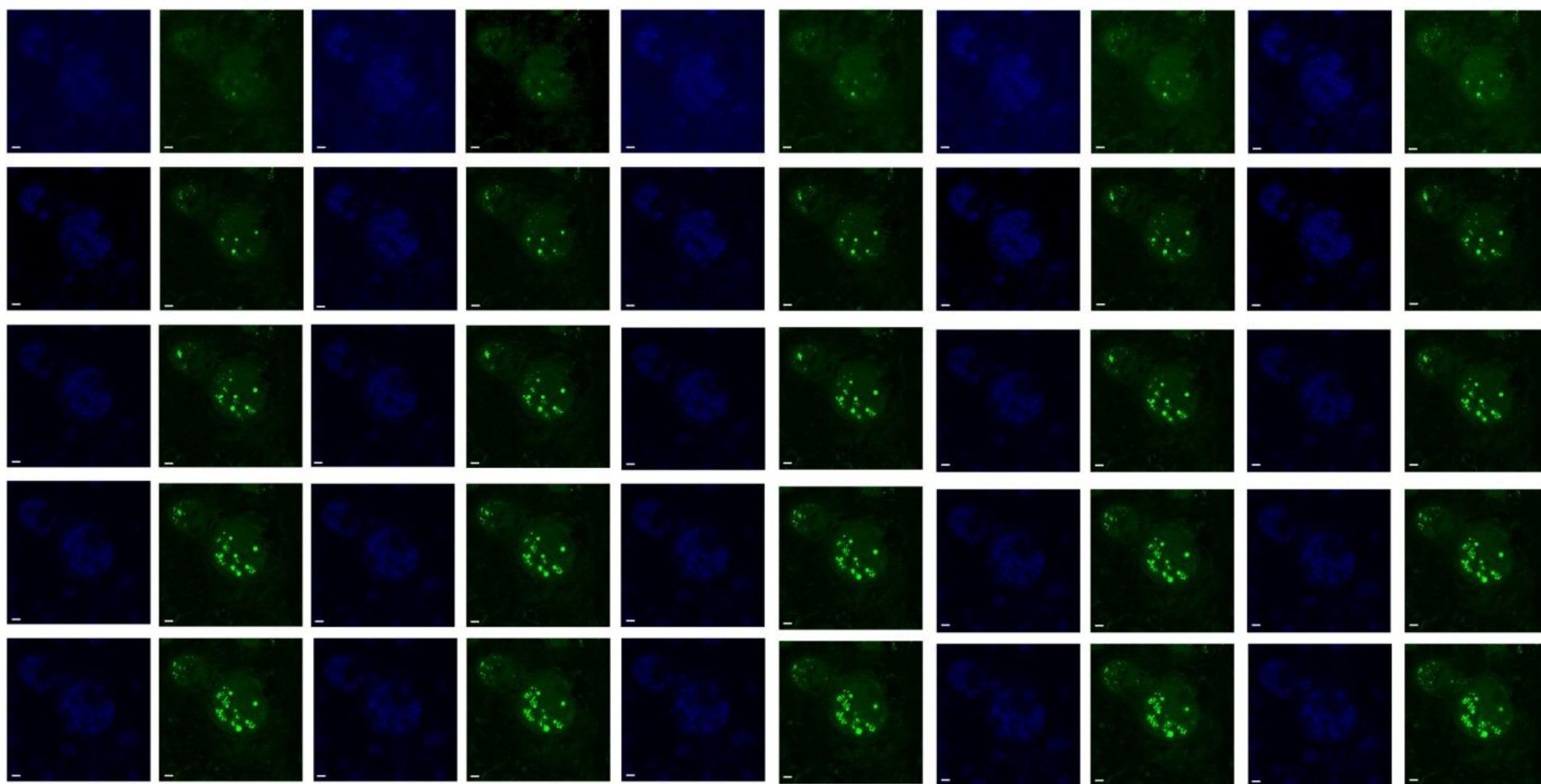

Supplement: Additional file 1: — Confocal sections of lipid droplet accumulation in a macromeront. 25 confocal sections of the composite Z-stack shown in Figure 3E are depicted. Nuclei were stained by DAPI (blue) whilst lipid droplets were shown in green by Bodipy 493/503 staining. Scale bars represent: 10 μm. [file 13567_2015_230_MOESM1_ESM.pdf]
